# Emerging oxidized and defective phases in low-dimensional CrCl_3_†

**DOI:** 10.1039/d1na00401h

**Published:** 2021-06-23

**Authors:** Dario Mastrippolito, Luca Ottaviano, Jing Wang, Jinjin Yang, Faming Gao, Mushtaq Ali, Gianluca D'Olimpio, Antonio Politano, Stefano Palleschi, Shafaq Kazim, Roberto Gunnella, Andrea Di Cicco, Anna Sgarlata, Judyta Strychalska-Nowak, Tomasz Klimczuk, Robert Joseph Cava, Luca Lozzi, Gianni Profeta

**Affiliations:** Department of Physical and Chemical Sciences, University of L'Aquila Via Vetoio 10 67100 L'Aquila Italy dario.mastrippolito@graduate.univaq.it; CNR-SPIN L'Aquila Via Vetoio 10 67100 L'Aquila Italy; Key Laboratory of Applied Chemistry, Yanshan University Qinhuangdao 066004 P. R. China; CNR-IMM Institute for Microelectronics and Microsystems VIII Strada 5 95121 Catania Italy; School of Science and Technology Physics Division, University of Camerino Italy; Department of Physics, Tor Vergata University of Rome Via Della Ricerca Scientifica 1 00133 Roma Italy; Faculty of Applied Physics and Mathematics, Gdansk University of Technology Gdansk Poland; Department of Chemistry, Princeton University Princeton NJ 08544 USA

## Abstract

Two-dimensional (2D) magnets such as chromium trihalides CrX_3_ (X = I, Br, Cl) represent a frontier for spintronics applications and, in particular, CrCl_3_ has attracted research interest due its relative stability under ambient conditions without rapid degradation, as opposed to CrI_3_. Herein, mechanically exfoliated CrCl_3_ flakes are characterized at the atomic scale and the electronic structures of pristine, oxidized, and defective monolayer CrCl_3_ phases are investigated employing density functional theory (DFT) calculations, scanning tunneling spectroscopy (STS), core level X-ray photoemission spectroscopy (XPS), and valence band XPS and ultraviolet photoemission spectroscopy (UPS). As revealed by atomically resolved transmission electron microscopy (TEM) and energy dispersive X-ray (EDX) analysis, the CrCl_3_ flakes show spontaneous surface oxidation upon air exposure with an extrinsic long-range ordered oxidized O–CrCl_3_ structure and amorphous chromium oxide formation on the edges of the flakes. XPS proves that CrCl_3_ is thermally stable up to 200 °C having intrinsically Cl vacancy-defects whose concentration is tunable *via* thermal annealing up to 400 °C. DFT calculations, supported by experimental valence band analysis, indicate that pure monolayer (ML) CrCl_3_ is an insulator with a band gap of 2.6 eV, while the electronic structures of oxidized and Cl defective phases of ML CrCl_3_, extrinsically emerging in exfoliated CrCl_3_ flakes, show in-gap spin-polarized states and relevant modifications of the electronic band structures.

## Abstract 2

Emerging defect- and oxidation-induced extrinsic phases are discovered in low-dimensional CrCl_3_.

## Graphical abstract

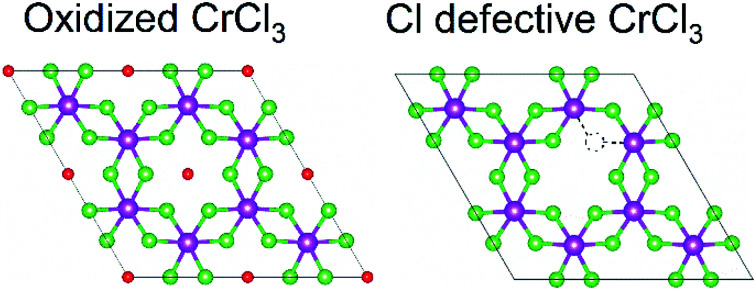

## 1 Introduction

Spintronics is an interdisciplinary field that merges materials science, condensed matter physics, and electronic engineering, aiming to simultaneously control both spin and charge degrees of freedom in nano-electronics.^1–3^ In the last two decades, it has dictated the road-map toward the fabrication of semiconducting magnetic materials with reduced dimensionality.^1,2^ Consequently, the efforts in the synthesis of new materials have moved from bulk^4,5^ toward two-dimensional (2D) materials, especially after the recent breakthrough discoveries of 2D magnetic systems.^6,7^ Among layered materials exhibiting magnetism in the bulk form and down to the monolayer limit, the class of chromium trihalides plays a central role.^8^ Cr-Trihalides (CrX_3_, X = I, Br, Cl) are van der Waals (vdW) materials exhibiting both insulating or semiconducting behavior and either ferro- or anti-ferromagnetism in their bulk phase.^9^ Research interest has primarily focused on few-layer CrI_3_,^10^ but its rapid degradation upon air exposure,^11^ which limits device fabrication, moved the attention towards CrCl_3_.^8,12–16^ Interestingly, CrCl_3_ can be experimentally studied even in reduced dimensions, as it easily exfoliates into large flakes down to the monolayer limit, starting from the bulk material and remaining stable under ambient conditions without rapid degradation.^17^ This enables real applications and multiple experimental investigations of CrCl_3_ in reduced dimensions. However, real applications face possible air exposure, degradation and the presence of defects, which so far have received little attention in Cr-trihalides. Recently, Grönke *et al.*^18^ found traces of oxygen attributable to surface oxidation of exfoliated CrCl_3_ flakes during transport in air, but it is not established whether flakes are really prone to oxidation upon air exposure and what are the possible effects of oxygen on the electronic structure.^6,7,19^ On the other hand, 2D vdW materials have native structural vacancy-defects^20^ that can be exploited by processes such as thermal annealing.^21^ Some theoretical studies on Cr-trihalides highlighted the role of defects in determining the electronic structure.^22–27^ Specifically, when Cr vacancy-defects are considered, transition to a half-metal is observed in all Cr-trihalides,^22,24,27^ while Cl vacancies narrow the band gap, as found in YCl_3_.^23^

In this study, we investigate the defective CrCl_3_ surface and experimentally demonstrate the existence of surface ordered oxidized and Cl defective phases of CrCl_3_ elucidating their role in the electronic structure. Our results show that mechanically exfoliated CrCl_3_ flakes, having a thickness of the order of tens of nanometers, show ordered surface oxidation upon air exposure and controllable Cl deficiency, remaining thermally stable up to 200 °C. The electronic structures of oxidized and Cl defective phases of monolayer (ML) CrCl_3_, extrinsically emerging in exfoliated CrCl_3_ flakes, show in-gap spin-polarized states and relevant modifications of the electronic bands.

## 2 Experimental and computational details

### 2.1 Experimental methods

CrCl_3_ crystals were grown *via* the self-transport technique starting from commercially available CrCl_3_ powders. The CrCl_3_ powder was first subjected to oxygen purification cycles and then sealed in a 20 cm long quartz glass tube and placed in a three-zone furnace with a temperature gradient of approximately 25 °C between the hot and the cold zones. The hot end of the tube was heated at 1000 °C, kept at this temperature for 1 hour, and then slowly cooled down to 700 °C at a 3 °C per hour cooling rate. The quartz tube was then held at 700 °C for 7 days. Crystals were stored and manipulated in a glove box with an argon atmosphere. Crystals were mechanically exfoliated in a glove box using an optimized scotch tape method.^17,28,29^

Transmission electron microscopy (TEM) experiments have been carried out with a TECNAI G2 TF30 STEM system transferring the CrCl_3_ flakes onto 200 mesh formvar grids. Low- and high-resolution (atomic) TEM images were acquired under bright field conditions. Diffraction patterns were acquired in diffraction mode. Elemental analysis has been carried out in scanning TEM mode using EDX spectroscopy (Oxford X-Max detector) and data have been quantitatively analyzed with the Cliff–Lorimer method.

Scanning tunneling spectroscopy (STS) measurements of air-exposed CrCl_3_ flakes were carried out in an UHV chamber at room temperature (RT) employing a scanning tunneling microscope (STM) Omicron VT-STM System with electrochemically etched W tips.^30^ Tunneling current–voltage (*I*–*V*) curves were acquired with STM in constant current mode (at −2 V of bias voltage offset).

X-ray photoemission spectroscopy (XPS) and ultraviolet photoemission spectroscopy (UPS) experiments were carried out in a ultra-high vacuum (UHV) chamber at RT on air-exposed and UHV annealed CrCl_3_ deposited onto 10 nm Au/Si(111) substrates. Surface sensitive angle integrated UPS spectra were measured using a He–I discharge lamp (*hν* = 21.2 eV) with the analyzer pass energy set to 5.85 eV. Correspondingly, XPS (*hν* = 1486.6 eV) VB spectra were acquired, for less surface sensitive VB analysis, with a PHI 1257 spectrometer with a monochromatic Al K_α_ source (*hν* = 1486.6 eV). The analyzer was operated at a spectral resolution of 100 meV, using a pass energy of 11.75 eV.

VB spectra were analyzed after Shirley background subtraction and calibrated with respect to experimental Fermi edges.

O 1s, Cr 2p_3/2_, and Cl 2p core levels were analyzed after Shirley background subtraction and calibration to the C 1s (284.5 eV) binding energy (signal from residual surface carbon contaminants). In the fitting procedure, spectra were decomposed using the sum of Lorentzian–Gaussian line-shapes with a fixed spin–orbit splitting of 9.2 eV (1.6 eV) for the Cr 2p (Cl 2p) doublets,^31^ keeping the full width at half maximum (FWHM) fixed for each spin–orbit doublet.

### 2.2 Computational details

Density functional theory (DFT) calculations were carried out using the Vienna *ab initio* simulation package (VASP) code,^32^ based on the projector augmented wave (PAW) method^33^ using the Perdew–Burke–Ernzerhof (PBE) exchange–correlation functional within the generalized gradient approximation (GGA) and considering vdW interactions using the semi-empirical dispersion-corrected density functional theory (DFT-D2) force-field approach.^34^ A 500 eV cut-off was considered for the kinetic energy and Monkhorst–Pack *k*-mesh for the Brillouin zone integration ensuring converged (1.0 × 10^−5^ eV per atom) total energies and residual forces (0.01 eV Å^−1^) after atomic relaxation.

The supercell approach was used to disentangle ML electronic structures using a vacuum region of 15 Å. Different concentrations of Cl vacancies were considered using 3 × 3, 2 × 2, and 2 × 1 supercells, accounting for Cl vacancy concentrations of 1.85%, 4.16%, and 8.33%, respectively.

## 3 Results and discussion

**Fig. 1:**
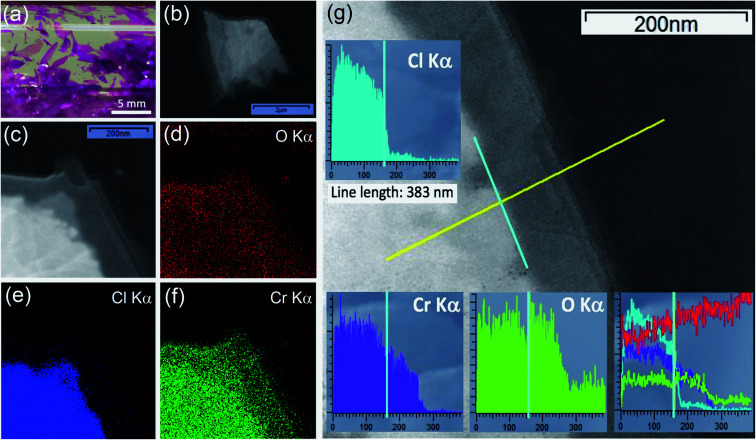
Characterization of mechanically exfoliated CrCl_3_ flakes. (a) Photograph of the as-grown CrCl_3_ crystals inside a quartz tube. Low-resolution TEM image of an air-exposed CrCl_3_ flake (b) and zoom in of the edge corner of the same flake (c). EDX maps of O K_α_ (d), Cl K_α_ (e), and Cr K_α_ (f) are focused on the same edge corner of the CrCl_3_ flake of panel (c). Results of EDX line scan (383 nm) analysis over one edge of the CrCl_3_ flake (g). The background greyscale image shows the TEM micrograph. The yellow line marks where the EDX line scan data are taken. The insets show EDX analysis for Cl K_α_ (cyan), Cr K_α_ (violet) and O K_α_ (light green) signals. The vertical intensity scales are in arbitrary units (bottom right inset). Direct comparison of EDX line scan signals including the C K_α_ signal (red), which is present on the TEM carbon grid. The cyan vertical line is drawn in the TEM micrograph and all insets in correspondence of 160 nm along the line scans, where the crystallographic step edge between the “bulk” and “few-layer” phase of flake is observed.

CrCl_3_ crystals were grown *via* a self-transport technique in a form of shiny and purple flakes, having a lamellar shape, with typically few millimeters lateral dimension (Fig. 1a).

In our previous study,^17^ identically synthesized crystals have been studied and characterized by X-ray diffraction (XRD), optical microscopy, atomic force microscopy, and micro-Raman spectroscopy and as-grown crystals turned out to be single-crystalline with a monoclinic *C*_2/*m*_ space group.^35^ Different from what was reported for other layered Cr-trihalides like CrI_3_, which is extremely unstable in air exhibiting immediate degradation,^11^ we have verified that there is no evident morphological degradation of CrCl_3_ flakes at the sub-micron level upon air exposure for tens of hours.^17^

Low-resolution TEM images (Fig. 1b and c) of an air-exposed CrCl_3_ flake confirm the lamellar structure of CrCl_3_, with a few-layer (estimated to be composed of 5–8 layers) contour region of lateral dimension of 100–200 nm, while the inner region can be identified as a “bulk-like” CrCl_3_ flake with the thickness in a range of 5–10 nm.^16^ As revealed by EDX maps of O K_α_, Cl K_α_, and Cr K_α_ (Fig. 1d–f), apart from the ubiquitous presence of carbon (C K_α_ map is not reported), the outer few-layer contour of the flake is characterized by the presence of Cr and O, while Cl is residual. This is confirmed by Fig. 1g, where the EDX signals are measured along the yellow line scan in the TEM image crossing the bulk/contour (few-layer/substrate grid) region. The three (Cl, Cr, and O) EDX line scans are also summarized, for a direct quantitative comparison, in the lower right inset of the same figure after the respective cross-sectional calibration, according to the Cliff–Lorimer method. The Cl/Cr/O ratio is approximately 3/2/1 in the inner bulk area of the flake, while, beyond the bulk edge, Cl drops by a factor of 15 to a residual signal and Cr notably survives with a Cr/O ratio close to unity. This result evidences the degradation of few-layer CrCl_3_ very likely into a chromium oxide phase, under ambient conditions and room temperature (RT). We have reason to believe that, to achieve such high oxidation levels, the exposure of both sides of the flakes to ambient conditions is crucial. Indeed, these findings are in line with recent reports,^36,37^ showing that CrCl_3_ acts as a precursor for mass production of 2D amorphous Cr_2_O_3_. However, at variance with these studies, where a rapid thermal exfoliation of CrCl_3_–6H_2_O was proposed, we demonstrate here that the transformation can occur even at RT and under ambient conditions.

### 3.1 Ordered oxidized O–CrCl_3_ phase

**Fig. 2:**
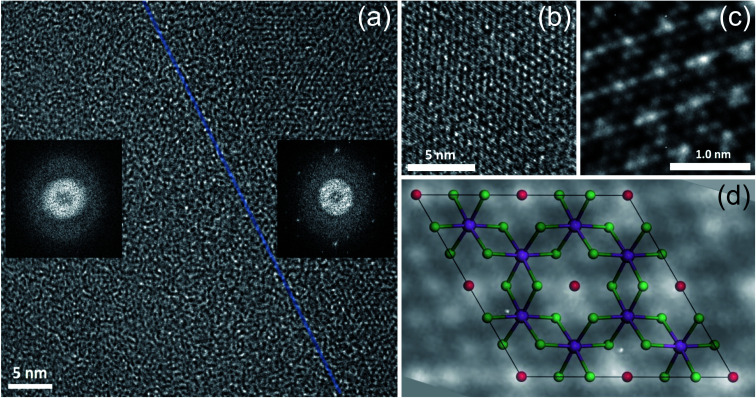
High-resolution TEM image acquired at the boundary between a few-layer/bulk region on a CrCl_3_ flake. The insets in panel (a) show fast Fourier transforms relative to the amorphous (left) and ordered (right) surface regions. A light blue semitransparent line has been artificially superimposed to the TEM data to mark the boundary between the two regions. High-resolution TEM images acquired at a different magnification of 590k× (b) and 1000k× (c), over the right ordered area of panel (a). (d) O–CrCl_3_ model structure superimposed to a detail of the frame of panel (c). Cr, Cl, and O atoms are represented in violet, green, and red spheres, respectively.

The same flake has been investigated in both the bulk and in the bulk/few-layer edge, acquiring high-resolution TEM (HRTEM) images (Fig. 2). The surface is characterized by two types of patterns and highlighted by a dividing light blue line in Fig. 2a. On the right side, we observe an ordered triangular lattice with an estimated lattice constant of 5.92 ± 0.07 Å. On the left side, in contrast, we find an amorphous oxidized region, systematically found in the contour regions of the flake (Cl deficient), lacking any long-range order. The assignments are based on the diffraction pattern of the different patches (insets of Fig. 2a). Different from the amorphous region, the ordered patch clearly shows the appearance of six hexagonal spots in the diffraction pattern. The ordered region is investigated more deeply with higher resolution HRTEM images at the atomic scale (Fig. 2b and c). In Fig. 2c, the typical atomic hexagonal structure of CrCl_3_ is recognizable, apart from the systematic presence of bright spots in the centers of the Cl hexagonal rings. We interpret these to originate from atoms sitting in the hollow sites of the CrCl_3_ plane with six Cl (Cr) as first (second) nearest neighbor atoms, still resulting in a (1 × 1) reconstruction of the surface. From the EDX elemental analysis, it's more than plausible that the atoms are oxygen. In Fig. 2d, we superimpose the HRTEM frame of Fig. 2c with a model structure of a ML CrCl_3_ with O atoms. It is evident that the brightest spots correspond to O atoms and cannot be confused with Cr or Cl atoms, showing reduced brightness. Thus, we found that the surface layer of CrCl_3_ forms an extrinsic ordered (1 × 1)-O–CrCl_3_ structure.

**Fig. 3:**
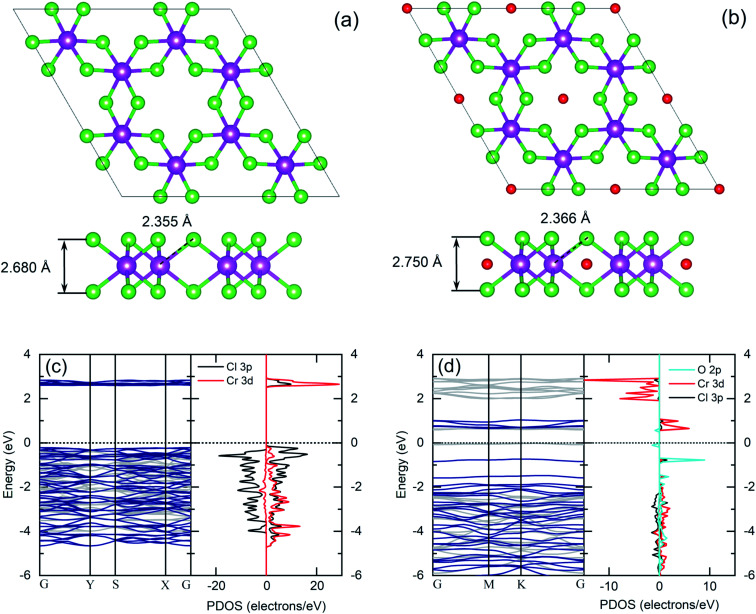
Top view and side view of crystal structures of pure monolayer CrCl_3_ (a) and O–CrCl_3_ structures (b). Cr, Cl, and O atoms are represented in violet, green, and red spheres, respectively. DFT GGA + *U* spin-resolved electronic band structures (left panels: light gray for spin-down and dark blue for spin-up) and projected density of states (right panels: left for spin-down and right for spin-up) of pure monolayer CrCl_3_ (c) and O–CrCl_3_ structures (d).

The experimental results of TEM and EDX analysis can be further validated by first-principles theoretical modeling of the oxidized surface, to understand the effect of O atoms on the electronic properties of CrCl_3_. In this section, we report the results of DFT calculations on pure ML CrCl_3_ and on ML O–CrCl_3_ model structures, emerging upon air exposure of CrCl_3_. To simulate the oxidized ordered O–CrCl_3_ phase, oxygen adsorption is considered in the hollow site formed by the six-member Cl ring, similar to the model of Li adsorption in CrI_3_.^38^ In line with that previously proposed,^22^ a reliable description of the electronic structure of Cr-halides can only be obtained within a GGA + *U* DFT approach, with on-site Coulomb repulsion, *U* = 5.0 eV on the Cr site (refer to the ESI† for pure GGA results, which provides the same qualitative conclusions). We modeled the systems using a (1 × 1) CrCl_3_ unit cell for both the clean and oxidized surface, in line with TEM experimental observations. The crystal structure of optimized pure ML CrCl_3_ and O–CrCl_3_ phases are shown in Fig. 3 with the corresponding spin-resolved electronic band structures and projected density of states (PDOS).

By total energy minimization for the pure ML CrCl_3_, we have calculated the in-plane lattice parameter (5.89 Å) which is close to the one reported for the bulk phase (space group *C*_2/*m*_).^9^ The internal parameters are the distance between the Cl planes of the sandwiched CrCl_3_ trilayer (2.68 Å) and the Cr–Cl bond length (2.35 Å) with a Cl–Cr–Cl bond angle of 90.0°. The oxidized phase is slightly modified with respect to the pure one. In particular, oxygen intercalates into the CrCl_3_ trilayer structure, sitting on the Cr atomic layer. This process is favored by the peculiar large hole in the surface Cl layer and increases the in-plane lattice parameter (6.13 Å) and both the Cl–Cl distance and the Cr–Cl bond lengths, resulting in 2.75 Å and 2.37 Å, respectively. Due to symmetry reduction, we find two different Cl–Cr–Cl bond angles of 98.81° and 83.06°. The O–Cl distance is 2.69 Å (see Table S1 of the ESI† for all the numerical values of optimized structural parameters). We now discuss the electronics properties. CrCl_3_ is an insulator with a wide band gap (2.63 eV) between the Cl 3p occupied spin-up (majority) state and the Cr 3d empty spin-up states. Interestingly, the first conduction band (CB) state is totally spin-up polarized. The electronic band structure of the oxidized ML CrCl_3_ is significantly modified. Oxygen hybridizes with all the valence band (VB) states and induces high localized in-gap spin-polarized O 2p states, reducing the band gap, which is now 0.67 eV based on DFT calculations. The Fermi level is pinned on the spin-down polarized O 2p states and the first CB of Cr 3d and Cl 3p states remain spin-up polarized, while the second CB is mainly formed by Cr 3d spin-down states.

**Fig. 4:**
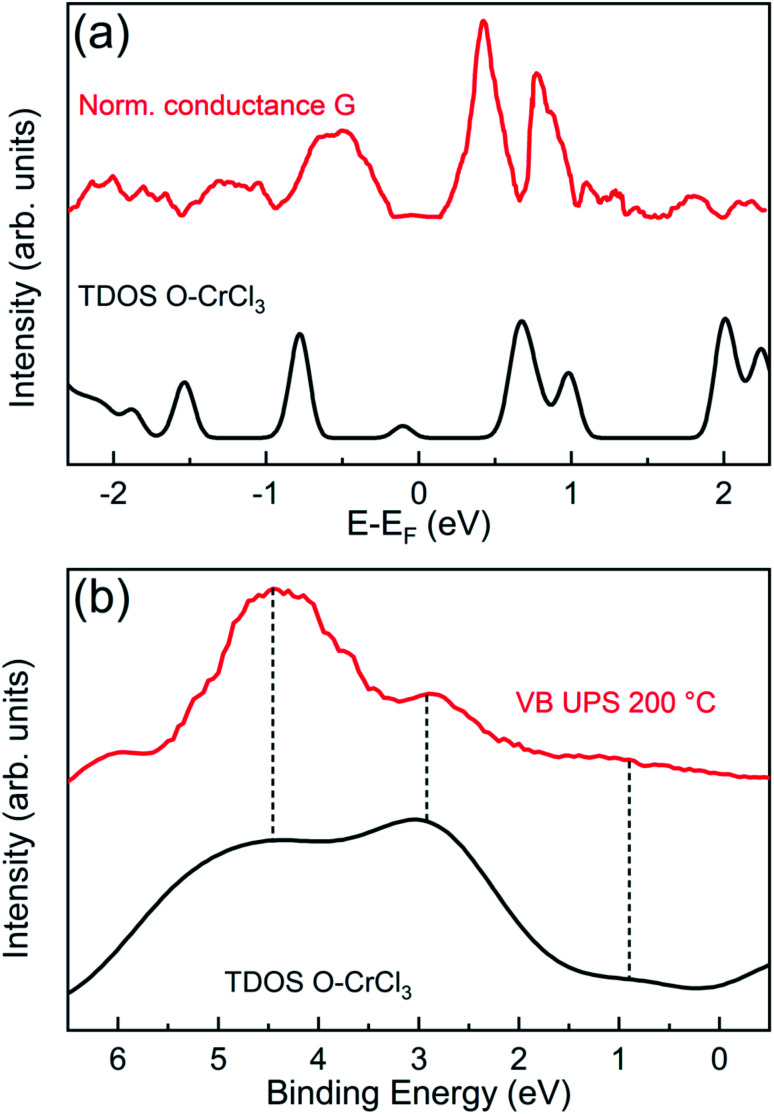
(a) GGA + *U* total density of states (convoluted with a Gaussian function σ = 0.05 eV) of the O–CrCl_3_ structure (black) are compared with mean normalized conductance (red) acquired on the surface of an air-exposed mechanically exfoliated CrCl_3_ flake. (b) UPS normalized valence band spectrum of the 200 °C UHV annealed CrCl_3_ sample (red) is compared to the GGA + *U* total density of states (convoluted with a Gaussian function σ = 0.5 eV) of the O–CrCl_3_ structure (black).

In order to validate our structural model and the theoretical predictions on the electronic properties, we performed surface sensitive STS and UPS and directly compared with GGA + *U* calculated total DOS of the O–CrCl_3_ phase (Fig. 4). The normalized conductance was calculated from experimental STS *I*–*V* curves, acquired at RT on the surface of air-exposed CrCl_3_ flake. The normalized conductance is defined as *G* = (d*I*/d*V*)/(*I*/*V*), where V is the sample bias voltage and I is the tunneling current. The mean normalized conductance curve, which mimics the surface DOS,^39^ was averaged over 15 different random locations on the surface of the CrCl_3_ flake. The calculated total DOS is convoluted with a Gaussian function (σ = 0.05 eV) to account for experimental resolution and thermal broadening effects at RT. The the measured and calculated DOS are in very good agreement for both occupied and empty states around the Fermi level. Interestingly, experiments confirm the presence of the induced in-gap O 2p state (close to −1 eV) and the presence of Cr 3d and Cl 3p states (close to 1 eV), predicted by calculations and clearly detected by STS measurements.

Independent measurements of surface states below the Fermi energy can be obtained by angle integrated UPS experiments to access the VB spectra of oxidized CrCl_3_, using a surface sensitive photon energy (*h*ν = 21.2 eV). The VB spectrum of the 200 °C UHV annealed CrCl_3_ flake, after air exposure, is compared with the GGA + *U* total DOS of the O–CrCl_3_ model structure, convoluted with a Gaussian function (σ = 0.5 eV). The predicted emerging in-gap O 2p state is clearly detected in the VB spectrum below the VB maximum (VBM) around 0.9 eV binding energy. The other experimental spectral features above 2 eV binding energy originate from hybridized O 2p, Cl 3p, and Cr 3d states and are reproduced by the calculated total DOS, showing two spectral features around 3 eV and 4.5 eV. The experimental evidence obtained from VB UPS, STS normalized conductance, and TEM analysis strongly support the proposed O–CrCl_3_ model structure as the emerging phase after air exposure of exfoliated CrCl_3_ flakes.

### 3.2 Cl defective CrCl_3_ phases

As stated before, phase stability upon air exposure and thermal treatment is crucial to determine electronic properties due to induced contaminants and defect generation. We thus characterized the chemical composition of the CrCl_3_ surfaces, investigating the temperature stability of the surface evolution of core levels by means of X-ray photoemission spectroscopy (XPS) experiments. All XPS data have been acquired at RT, using an Al K_α_ source (hν = 1486.6 eV), onto CrCl_3_ flakes mechanically exfoliated and inserted in UHV at RT (hereafter referred to as 20 °C samples) and subsequently subjected to one hour of UHV thermal annealing at 100 °C, 200 °C, 300 °C, and 400 °C, respectively.

**Fig. 5:**
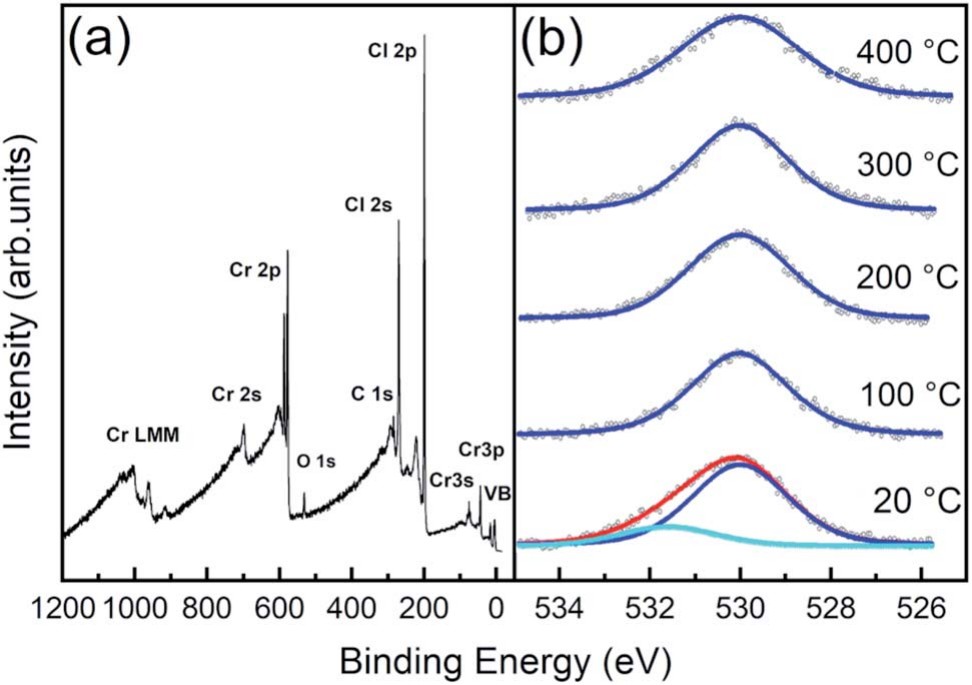
(a) XPS survey spectrum acquired at RT for a CrCl_3_ flake. (b) O 1s normalized core level fitted spectra acquired at RT and after one hour UHV thermal annealing at different temperatures.

In the survey spectrum of the 20 °C sample (Fig. 5a) and in all the other samples (apart from a minor C 1s contamination), only three main elemental signals were detected, corresponding to the core level excitations of Cr, Cl, and O. In particular, oxygen is not just present as a physisorbed surface contaminant, but we find it chemisorbed since it is still observed after all the post-high-temperature UHV annealing processes, as visible in O 1s spectra (Fig. 5b). Notably, the spectrum is easily decomposed into a main O 1s peak at 530.0 eV (blue curves), which is the typical binding energy of oxidized phases of chromium^37,40–44^ (corresponding to O^2−^),^40^ and an additional spectral component at 531.7 eV, assigned to OH and possible contaminants on the surface, since this component is removed after 100 °C UHV annealing.

The surface atomic concentration estimates of Cr, Cl, and O and the Cl/Cr ratio are reported in Table 1. For the analysis, the spectral intensities have been calculated considering the whole spectral areas of the Cr 2p_3/2_, Cl 2p, and O 1s line shapes. Surface sensitive atomic sensitivity factor (ASF) values of 0.770, and 0.711 were considered for Cl and O, respectively^31^ (the derivation of the corrected Cr ASF is discussed in the ESI†). Apart from a slight O desorption observed upon annealing at 100 °C, the system exhibits thermal stability up to 200 °C, with almost unchanged Cr concentration and slight increase (decrease) of Cl (O) with the Cl/Cr ratio ranging from 2.7 to 2.6. Then, at annealing temperatures above 200 °C, we observe progressive Cl desorption accompanied by slight O desorption, between 200 °C and 300 °C, with a corresponding increase of the Cr signal (the Cl/Cr ratio drops to 2.2).

**Table 1:** Summary table of the atomic concentrations (at%) and Cl/Cr ratio of CrCl_3_ samples at different annealing temperatures

*T* (°C)	Cl (%)	Cr (%)	O (%)	Cl/Cr ratio
20	68.8	25.6	5.6	2.69
100	69.0	26.2	4.8	2.63
200	68.9	26.4	4.7	2.60
300	67.5	28.8	3.7	2.35
400	66.3	30.2	3.5	2.20

**Table 2:** Summary table of the Cr 2p_3/2_ and Cl 2p fitted weight component evolution with annealing temperature

*T* (°C)	Cr 2p_3/2_ components						Cl 2p components			
	Cr^6+^	Cr^5+^	Cr^4+^	Cr^3+^	Cr^2+^	Cr^+^	Contaminants	CrCl_3_ main	Cl vacancies	Metallic Cl
	579.2 eV	578.7 eV	578.0 eV	577.0 eV	575.7 eV	574.4 eV	200.3 eV	199.5 eV	198.2 eV	197.0 eV
20	0.06	0.10	0.35	0.42	0.07	—	0.10	0.78	0.12	—
100	—	0.11	0.27	0.48	0.14	—	—	0.83	0.16	—
200	—	0.11	0.25	0.46	0.18	—	—	0.84	0.16	—
300	—	—	0.15	0.35	0.33	0.17	—	0.68	0.23	0.08
400	—	—	0.11	0.28	0.36	0.23	—	0.52	0.34	0.13

**Fig. 6:**
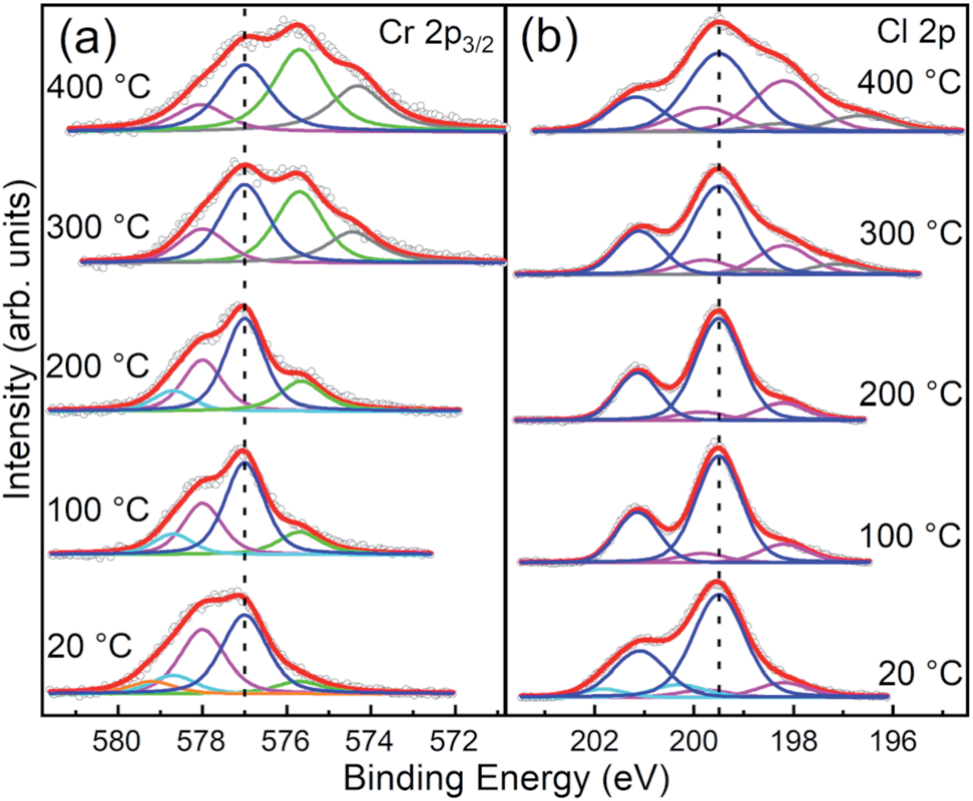
Normalized fitted spectra of Cr 2p_3/2_ and Cl 2p core levels of CrCl_3_ flakes acquired at RT and after UHV thermal annealing (1 hour) at 100 °C, 200 °C, 300 °C, and 400 °C. The raw data (empty black circles) and the cumulative fits (red) are shown. (a) Cr 2p_3/2_ spectra are fitted with Cr^6+^ (orange), Cr^5+^ (cyan), Cr^4+^ (magenta), Cr^3+^ (blue), Cr^2+^ (green), and Cr^+^ (gray) components. (b) Cl 2p spectra are fitted with contamination (cyan), main (blue), Cl vacancies (magenta), and metallic Cl (gray) components. See Table 2 for typical energy position and relative weights of each components.

The evolution of the Cr 2p_3/2_ and Cl 2p core level spectra with the annealing temperature is reported in Fig. 6a and b, respectively. Apart from a decrease of the broadening of the core levels upon 100 °C annealing, both Cr and Cl core level line shapes remain substantially unaltered up to 200 °C annealing temperature. Above 200 °C, consistent with the changes in the stoichiometry already discussed, the spectra show modifications of their line-shapes. Specifically, the Cr 2p_3/2_ line-shape is progressively modified towards the ones observed in Cr_2_O_3_,^37,40–42,44^ the most stable phase among chromium oxides.

Although the shape of the Cr 2p_3/2_ spectra resembles that reported in the literature for CrCl_3_ in a powder phase,^42^ we observe other components that cannot be assigned only to pure CrCl_3_ due to the presence of oxygen and the specific binding energy position of the O 1s core level of 530 eV that indicates the presence of chromium oxide. In addition, the Cl/Cr ratio is below the expected 3 : 1 ratio. Therefore, considering previous TEM analysis, we conclude that coexisting phases of CrCl_3_ and Cr_2_O_3_ are present. To strengthen this consideration, we compared our Cr 2p_3/2_ spectrum, acquired after 100 °C annealing, with the Cr 2p_3/2_ spectra of the CrCl_3_ powder^42^ and Cr_2_O_3_ (ref. 40 and 41) (Fig. S1 of ESI†) reported in the literature. The two core level line-shapes span essentially the same energy range and it is difficult to distinguish the Cr oxide related component from the Cr 2p_3/2_ line shape. To further complicate the scenario, deviation from the pure stoichiometry of the compounds under investigation should also to be considered. Indeed, Cr_2_O_3_ is prone to be both Cr and O deficient leading to the presence of possible sub-oxides.^40^ As a matter of fact, and for the sake of simplicity, the following analysis of the Cr 2p_3/2_ spectra must necessarily remain qualitative. For the stable phase up to 200 °C, we observe four main components at 575.7 eV, 577.0 eV, 578.0 eV, and 578.7 eV, assigned to different Cr oxidation states: Cr^2+^, Cr^3+^, Cr^4+^, and Cr^5+^, respectively.^41^ The most intense component, up to 300 °C UHV annealing temperature, is the Cr^3+^ one consistent with the octahedral coordination of the Cr^3+^ ions with Cl^−^. A significant increase of the Cr^2+^ and Cr^+^ components is observed with annealing temperature, simultaneously with the Cl desorption and the slight O desorption. We attribute this reduction of oxidation states to the introduction of Cl vacancy-defects. Indeed, we can exclude that this occurrence is due to emerging new chromium oxide phases, different from Cr_2_O_3_, since the O 1s peak position remains almost unchanged and no other components are detected even at 400 °C annealing temperature. Thus, in order to explore this possibility, we extend our XPS analysis to Cl 2p core levels which mainly provides information on the evolution of the CrCl_3_ phase and allows us to quantify the Cl vacancy formation. Moreover, different from Cr and apart from the spin–orbit splitting, the Cl 2p spectrum does not have multiplet structures. Therefore, various components can be assigned directly to chemical shift effects. At variance with that reported in the literature for the CrCl_3_ powders,^42^ the Cl 2p core level line-shape cannot be fitted considering a single doublet, but the inclusion of an additional doublet at a lower binding energy is required for all the spectra, and a further high binding energy doublet is required for the not annealed one. This last component is not needed after 100 °C annealing, and thus can be safely assigned to contaminants, in accordance with interpretation of the O 1s feature. In contrast, the additional low binding energy doublet is almost constant up to 200 °C and accounts for 16% of the overall spectral weight, and then it starts to increase up to 34% at 400 °C annealing temperature. We assign this component to Cl atoms surrounding Cl vacancies following a similar assignment as in ref. 21 for MoS_2_. Assuming a random distribution of Cl vacancies at a low concentration and considering that only the nearest neighbor Cl atoms surrounding the Cl vacancy are chemically affected, the measured 16% for the low binding energy component accounts for 2.3% of the Cl vacancy concentration. This is a very plausible estimation for the intrinsic vacancy concentration in vdW materials.^20,21^ According to this interpretation model, the Cl vacancy concentration grows up to 3.4% after annealing at 300 °C and reaches 5% after annealing at 400 °C. An additional lower binding energy doublet in Cl 2p spectra is observed after UHV annealing at a temperature above 300 °C, which can be assigned to the possible formation of metal chloride,^45^ which we labeled as “metallic Cl”. Indeed, for the XPS analysis, our flakes were deposited on metallic substrates (Au/Si(111)) and thus we cannot exclude high-temperature metal chloride formation, considering that Liu *et al.*^45^ observed metal chloride and CrCl_2_ formation *via* accelerated degradation on air-exposed mechanically exfoliated CrCl_3_ nanoflakes on the electrode interface. We observe a similar behavior, but with UHV high-temperature thermal annealing. However, the metal chloride and CrCl_2_ formation are consistent with Cl vacancies and in line with the relevant Cr^2+^ component observed in our Cr 2p_3/2_ spectra above 300 °C annealing temperature.

**Fig. 7:**
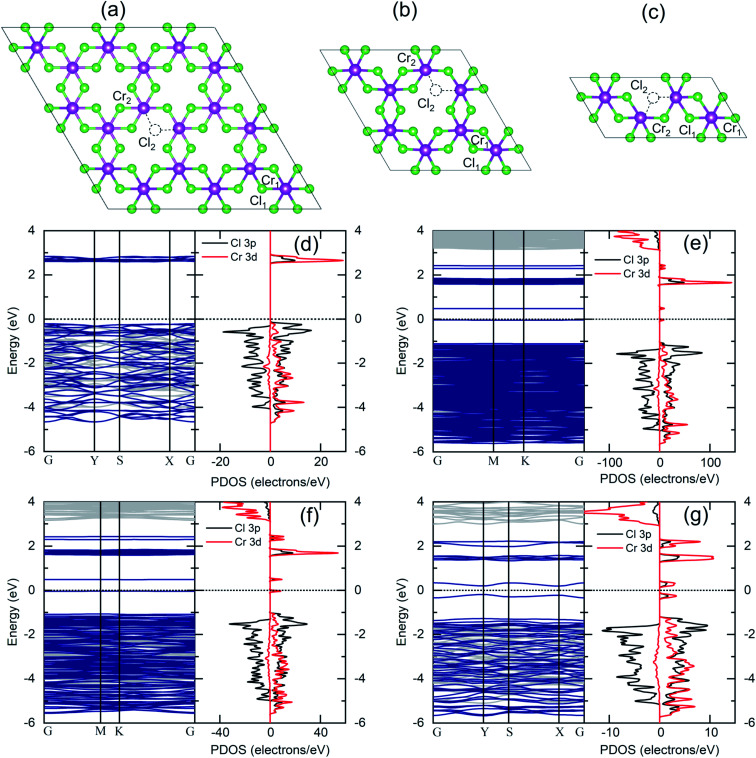
Top views of model structures of Cl defective monolayer CrCl_3_: 1.85% Cl defective (3 × 3 supercell) (a), 4.16% Cl defective (2 × 2 supercell) (b) and 8.33% Cl defective (2 × 1 supercell) (c). Cr and Cl atoms are represented in violet and green spheres, respectively. (d–g) DFT GGA + *U* spin-resolved electronic band structure (left panel: light gray for spin-down and dark blue for spin-up) and projected density of states (right panel: left for spin-down and right for spin-up) of pure monolayer CrCl_3_ (d) and Cl defective monolayer CrCl_3_, with increasing Cl vacancy concentrations of 1.85% (e), 4.16% (f), and 8.33% (g).

Given the outcomes of XPS analysis, a more realistic model structure for electronic structure calculations must take into account the much likely occurrence of Cl defective phases. We thus considered Cl deficient CrCl_3_ supercells (Fig. 7a–c) to simulate different Cl vacancy concentrations in a low range (1–10%). In addition to the pure ML CrCl_3_ phase, we considered different model structures containing one Cl vacancy: 3 × 3 supercell (a), 2 × 2 supercell (b), and 2 × 1 supercell (c) corresponding to 1.85%, 4.16%, and 8.33% of Cl vacancies, respectively. Considering Fig. 7, the Cr_1_ atom stands for the Cr atom without any nearest neighbor Cl vacancy, while Cr_2_ represents the Cr atom with a missing Cl nearest neighbor. Cl vacancies can be located in the two Cl sublattices, Cl_1_ or Cl_2_. For all the concentrations, we found a 1.0% expansion of the in-plane lattice parameters and a 5% relaxation of the vertical Cl–Cl distance between the Cl planes of the sandwiched CrCl_3_ trilayer. The Cl vacancy induces a slight distortion of the CrCl_3_ structures (the optimized structural parameters for pure and Cl defective ML CrCl_3_ are reported in Table S1 of the ESI†). Fig. 7 (panels d–g) shows the DFT GGA + *U* spin-resolved electronic band structures and PDOS of pure and Cl defective ML CrCl_3_ phases. Cl vacancies induce two in-gap spin-up polarized states originating from the Cr 3d orbitals of Cr_1_ and Cl 3p from the nearest neighbor Cl atoms of the vacancy. This leads to reduction of the band gap, similar to the transition observed in the oxidized phase. The calculated band gaps of Cl defective structures at different Cl vacancy concentrations are 0.53 eV for 1.85%, 0.52 eV for 4.16%, and 0.39 eV for 8.33%. We highlight that Cl vacancies, contrary to the Cr vacancy case, do not lead to a half-metal as observed in a previous study.^22^ The emerging in-gap states remain almost flat up to 4.16% Cl vacancy concentration, but at 8.33% start to disperse. The Fermi level is pinned at a high energy in the gap, resulting in an n-type doping induced by Cl vacancies.

**Fig. 8:**
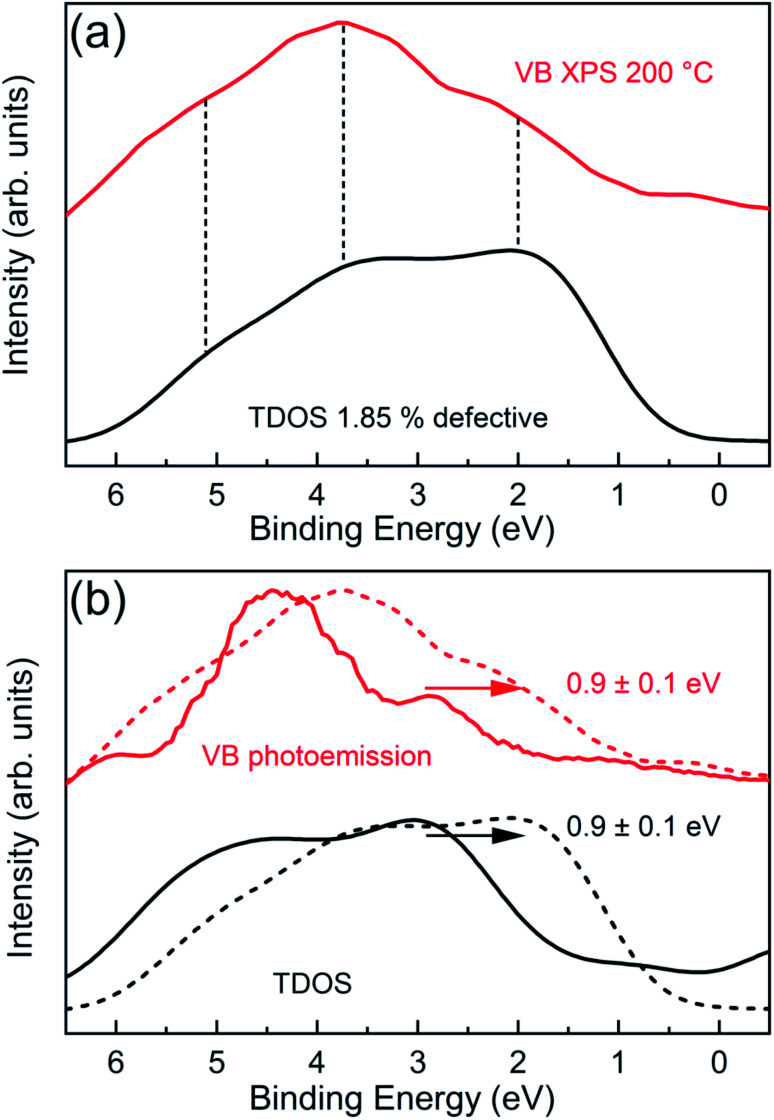
(a) XPS normalized valence band spectrum of the 200 °C UHV annealed CrCl_3_ flake (red) is compared to the GGA + *U* total density of states (convoluted with Gaussian function σ = 0.5 eV) for the 1.85% Cl defective CrCl_3_ structure. (b) Normalized valence bands of the 200 °C UHV annealed CrCl_3_ sample, acquired with surface sensitive UPS (red curve) and bulk sensitive XPS (dashed red curve), are directly compared with the GGA + *U* total density of states of O–CrCl_3_ (black curve) and the 1.85% Cl defective CrCl_3_ (dashed black curve) structure. The valence bands of the Cl defective phase are pinned of 0.9 ± 0.1 eV with respect to the oxidized phase.

To further confirm the DFT predictions on Cl vacancy induced effects, we have measured angle integrated VB XPS spectra of the CrCl_3_ flake using a bulk sensitive photon energy (*h*ν = 1486.7 eV). In this way, the contribution of surface oxidation is minimized and we can probe the inner layers of the CrCl_3_ flakes. Fig. 8a shows the experimental VB spectrum of the 200 °C UHV annealed CrCl_3_ sample, which has a Cl vacancy concentration of ≃2% as estimated by XPS analysis, compared with the total DOS of 1.85% Cl defective CrCl_3_ convoluted with a Gaussian function (σ = 0.5 eV). The most prominent experimental VB spectral features are around 2 eV, then at 3.7 eV, and 5.1 eV. The comparison with the theoretical total DOS highlights how all the characteristic features are predicted and can be interpreted as originating from Cl 3p states and hybridized Cl 3p and Cr 3d states, respectively. To further highlight the role of Cl vacancies with respect to surface oxidation, we report a complete summary of the VB spectra of both oxidized and Cl defective CrCl_3_ phases (Fig. 8b), including the respective GGA + *U* total DOS. As clearly visible, the VB states of the Cl defective phase are shifted by ≃0.9 eV with respect to the oxidized ones, which is perfectly accounted by first-principles calculations.

## 4 Conclusions

We characterized homegrown mechanically exfoliated CrCl_3_ flakes upon air exposure and investigated the electronic structures and properties of emerging surface oxidized and Cl defective phases by means of different techniques. The experimental results have been corroborated with DFT based calculations. In line with our previous findings,^17^ we confirm the relative stability of the CrCl_3_ flakes at the sub-micron scale and under ambient conditions, which do not undergo dramatic degradation effects, and show thermal stability up to 200 °C, making the system suitable for large scale applications. Under ambient conditions, atomic scale TEM combined with EDX elemental microanalysis indicates that few-layer CrCl_3_ flakes develop a surface amorphous chromium oxide on the edges of the flake and an inner extrinsic long-range ordered oxidized O–CrCl_3_ phase as being formed by the inclusion of one oxygen atom per unit cell in the hollow site at the Cr atomic layer. The X-ray core levels analysis indicates that CrCl_3_ flakes are under-stoichiometric exhibiting a few % of Cl vacancies, tunable by thermal annealing up to 400 °C.

First-principles calculations performed on pure, oxidized and Cl defective monolayer CrCl_3_ phases confirm that pure CrCl_3_ is an insulator, while the oxidized and Cl defective phases have emerging in-gap spin-polarized states. The electronic structure calculations are performed by surface sensitive STS and VB UPS analysis for both the surface oxidized phase and bulk sensitive VB XPS analysis for the Cl defective phase.

The outcomes of the present systematic experimental and theoretical investigation make CrCl_3_ a versatile candidate for spintronics and multifunctional nanodevices, and could be easily extended to other members of the trihalides class, which can further enrich the interesting physical phenomena they show. In particular, the two discovered phases could represent an additional ingredient that makes this class of 2D magnetic systems even more interesting. Indeed, considering the recent observation of topological excitation on ferromagnetic CrI_3_ (ref. 46) together with the possibility of spanning the magnetic phase diagram through the in-plane strain field,^47^ the doping effect^25^ and the field effect,^48^ the inclusion of the oxygen atom and Cl vacancy-defects can potentially enable the realization of new magnetic phases and excitations.

## Author contributions

Dario Mastrippolito: conceptualization, data curation, formal analysis, investigation, validation, visualization, writing – original draft, and writing – review & editing. Luca Ottaviano: conceptualization, formal analysis, funding acquisition, methodology, project administration, resources, supervision, and writing – review & editing. Jing Wang: formal analysis, resources, software, and validation. Jinjin Yang: formal analysis, software, and validation. Faming Gao: formal analysis, resources, software, and validation. Mushtaq Ali: data curation, formal analysis, investigation, validation, and writing – original draft. Gianluca D'Olimpio: investigation. Antonio Politano: conceptualization. Stefano Palleschi: investigation. Shafaq Kazim: investigation. Roberto Gunnella: formal analysis, conceptualization, and writing – review & editing. Andrea Di Cicco: conceptualization. Anna Sgarlata: investigation, resources. Judyta Strychalska-Nowak: investigation. Tomasz Klimczuk: conceptualization, investigation, and resources. Robert Joseph Cava: conceptualization. Luca Lozzi: investigation, resources. Gianni Profeta: conceptualization, formal analysis, funding acquisition, methodology, project administration, supervision, and writing – review & editing.

## Conflicts of interest

There are no conflicts to declare.

## Supplementary Material

NA-003-D1NA00401H-s001
