# Supplementary material for: Emerging oxidized and defective phases in low-dimensional CrCl3
Source: Nanoscale Adv. 2021 Jun 23;3(16):4756–66. doi: 10.1039/d1na00401h (PMC9419748; doi:10.1039/d1na00401h)
Supplement: NA-003-D1NA00401H-s001 [file NA-003-D1NA00401H-s001.pdf]

Electronic Supplementary Material (ESI) for Nanoscale Advances.
This journal is © The Royal Society of Chemistry 2021

Supplementary Information
Emerging oxidized and defective phases in low-dimensional $CrCl_3$

Dario Mastrippolito,[1,*] Luca Ottaviano,[1,2] Jing Wang,[3] Jinjin Yang,[3] Faming Gao,[3] Mushtaq Ali,[1] Gianluca D'Olimpio,[1] Antonio Politano,[1,4] Stefano Palleschi,[1] Shafaq Kazim,[5] Roberto Gunnella,[5] Andrea Di Cicco,[5] Anna Sgarlata,[6] Judyta Strychalska-Nowak,[7] Tomasz Klimczuk,[7] Robert Joseph Cava,[8] Luca Lozzi,[1] and Gianni Profeta[1,2]

[1]*Department of Physical and Chemical Sciences, University of L'Aquila, Via Vetoio 10, 67100 L'Aquila, Italy*
[2]*CNR-SPIN L'Aquila, Via Vetoio 10, 67100 L'Aquila, Italy*
[3]*Key Laboratory of Applied Chemistry, Yanshan University, Qinhuangdao, 066004, P.R. China*
[4]*CNR-IMM Institute for Microelectronics and Microsystems, VIII strada 5, 95121 Catania, Italy*
[5]*School of Science and Technology Physics division, University of Camerino, Italy*
[6]*Department of Physics, Tor Vergata University of Rome, Via Della Ricerca Scientifica 1, 00133 Roma, Italy*
[7]*Faculty of Applied Physics and Mathematics Gdansk University of Technology, Gdansk, Poland*
[8]*Department of Chemistry Princeton University, Princeton, NJ 08544, United States of America*

[*] email: dario.mastrippolito@graduate.univaq.it

I. CORE LEVEL PHOTOEMISSION

The core level spectra reported in the main article have been quantitatively analyzed to determine the relative Cl/Cr atomic ratio and the elemental surface concentration by considering the values of 0.770 and 0.711 of the atomic sensitivity factors as reported in Ref. [1]. The Cr atomic sensitivity factor has been recalculated by assuming the presence of Cr in two phases, namely $CrCl_3$ and Cr_2O_3. Under this assumption, the Cr $2p_{3/2}$ atomic sensitivity factor (ASF_{Cr}) can be estimated from the experimentally measured spectral area (A_{Cr}) of the Cr 2p core level with the following:

$$ASF_{Cr} = \frac{3A_{Cr}}{2A_O/0.711 + A_{Cl}/0.770},$$

where A_O and A_{Cl} are the experimental areas of O 1s and Cl 2p, respectively.

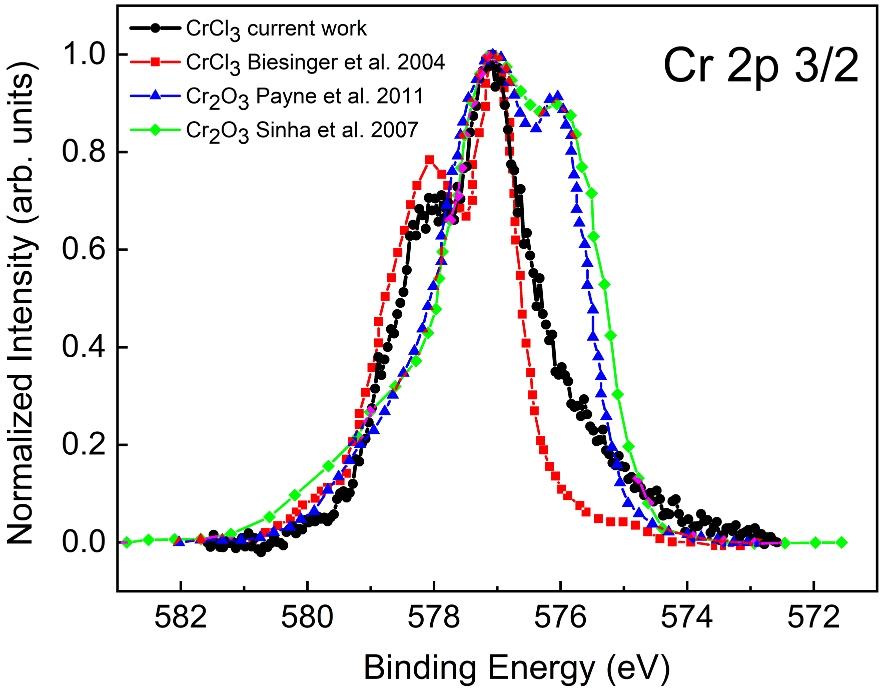

FIG. 1. Comparison with literature of normalized Cr $2p_{3/2}$ spectrum of this work (acquired after 1 hour 100 °C UHV annealing (black)). Powder phase $CrCl_3$ reference spectrum is digitized form Ref. [2] (red). Cr_2O_3 reference spectra are digitized from Ref. [3] (blue) and Ref. [4] (blue).

II. STRUCTURAL PARAMETERS

TABLE I. Optimized structural parameters for pure and oxidized $CrCl_3$ monolayers and Cl defective $CrCl_3$ supercells. Cr_1 is a Cr atom with an unperturbed (non defective) first neighboring shell of 6 Cl atoms (labeled as Cl_1), while Cr_2 atoms are those Cr ones in the structure which are first neighbors of a Cl vacant site (labeled as Cl_2).

	Pure	Oxidized	(3×3)	(2×2)	(2×1)
a (Å)	5.89	6.13	17.97	11.98	11.98
b (Å)	5.89	6.13	17.99	12.02	6.04
c (Å)	2.68	2.75	2.81	2.83	2.81
α (°)	90.0	83.1	90.0	89.6	89.2
β (°)	90.0	98.8	90.0	90.2	90.4
γ (°)	120.0	120.0	120.0	120.1	120.3
Cr_1-Cl (Å)	2.35x6	2.37x6	2.36x6	2.36x6	2.36x3
				2.36x2	
				2.37x1	
Cr_2-Cl (Å)	-	-	2.36x2	2.36x2	2.36x2
			2.33x1	2.33x1	2.33x1
			2.34x1	2.34x1	2.34x1
			2.34x1	2.34x1	2.35x1

III. ELECTRONIC STRUCTURES AND DENSITY OF STATES

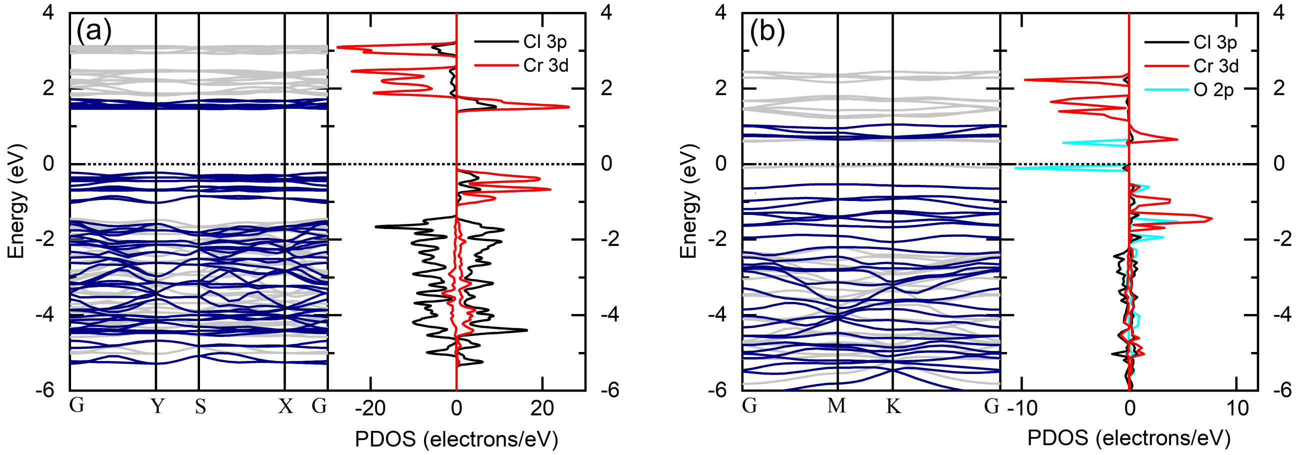

FIG. 2. DFT GGA spin-resolved electronic band structures (left panel: light gray for spin-down and dark blue for spin-up) and projected density of states (right panel: left for spin-down and right for spin-up) of the pure monolayer $CrCl_3$ (a) and O-$CrCl_3$ structure (b).

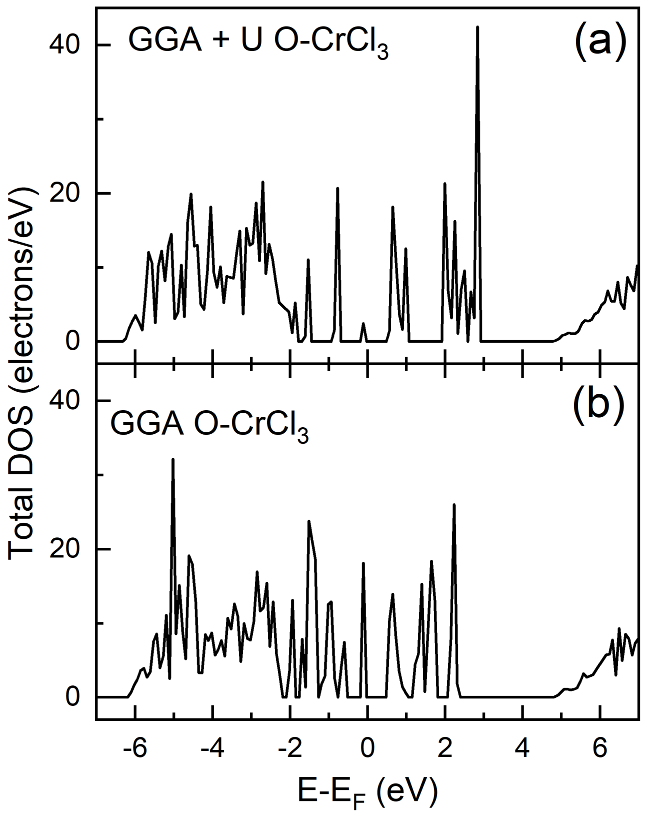

FIG. 3. Total density of states for O-$CrCl_3$ structures calculated with GGA + U (a) and GGA (b) approaches.

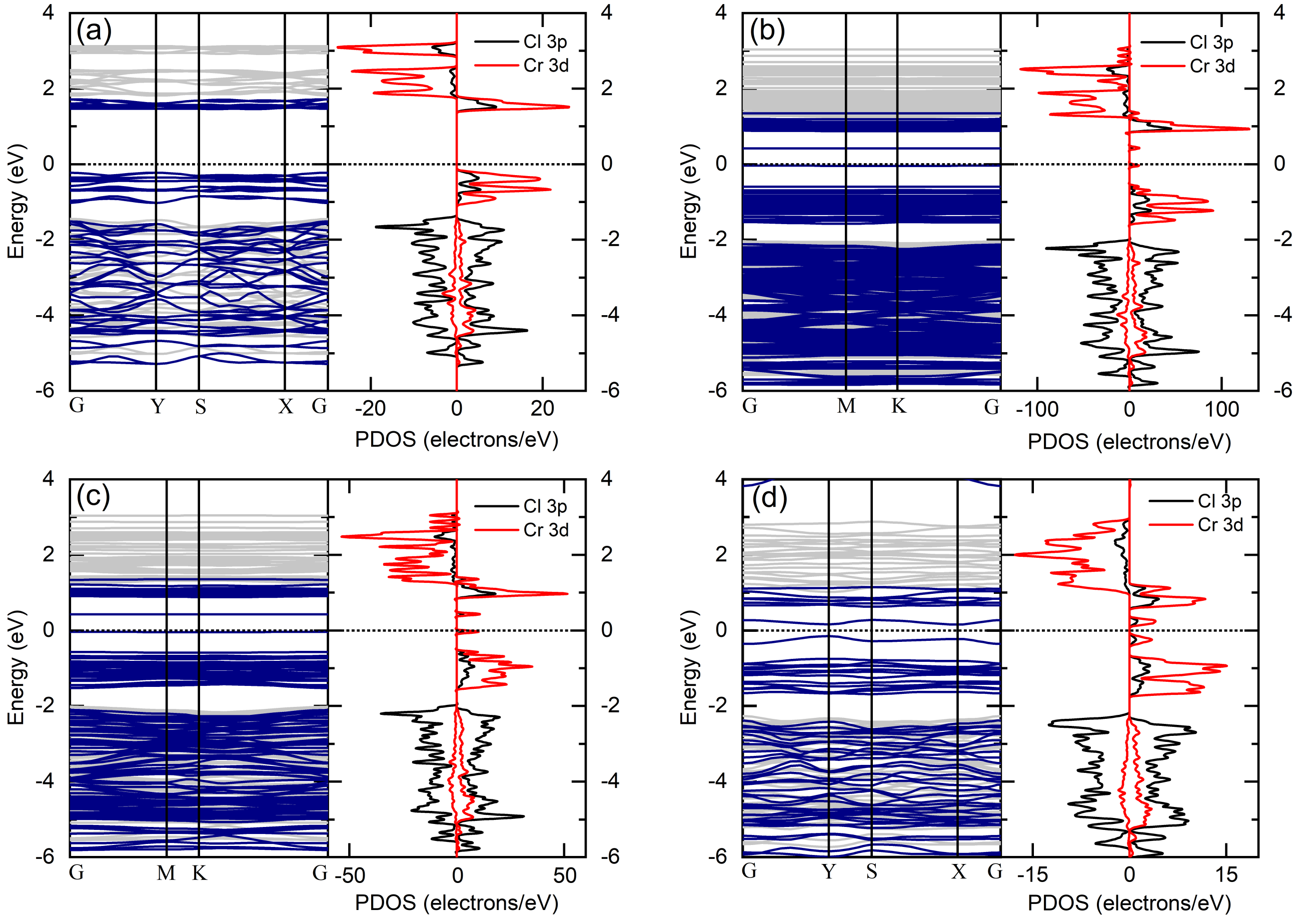

FIG. 4. DFT GGA spin-resolved electronic band structure (left panel: light gray for spin-down and dark blue for spin-up) and projected density of states (right panel: left for spin-down and right for spin-up) of pure monolayer $CrCl_3$ (a) and Cl defective monolayer $CrCl_3$ with increasing Cl vacancy concentrations of 1.85 % (b), 4.16 % (c), and 8.33 % (d).

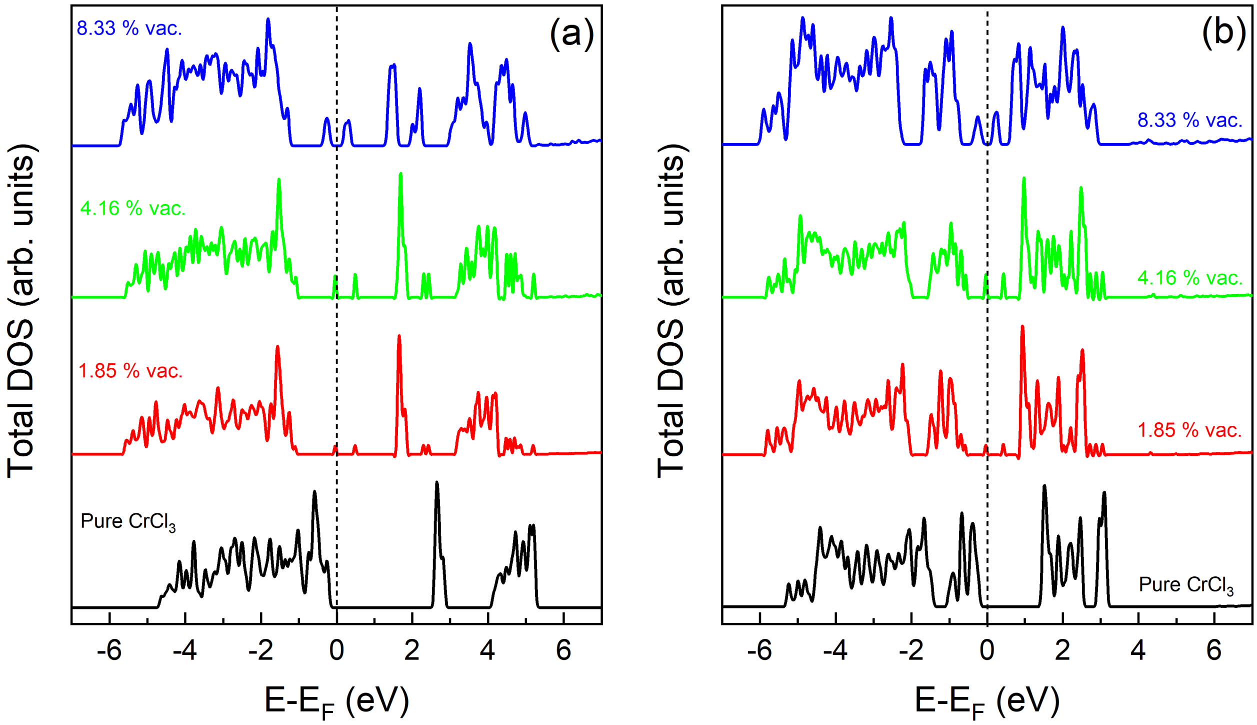

FIG. 5. Total density of states, calculated with GGA + U (a) and GGA (b) methods, for pure monolayer $CrCl_3$ (black) and Cl defective monolayer $CrCl_3$ structures: (3×3) 1.85 % Cl vacancy (red), (2×2) 4.16 % Cl vacancy (green), and (2×1) 8.33 % Cl vacancy (blue).

[1] J. F. Moulder, W. F. Stickle, P. E. Sobol, and K.D. Bomben, *Handbook of X-ray Photoelectron Spectroscopy: A Reference Book of Standard Spectra for Identification and Interpretation of XPS Data* (Physical Electronics Division, Perkin-Elmer Corporation, 1992).

[2] MC Biesinger, C Brown, JR Mycroft, RD Davidson, and NS McIntyre, "X-ray photoelectron spectroscopy studies of chromium compounds," Surface and Interface Analysis **36**, 1550–1563 (2004).

[3] BP Payne, MC Biesinger, and NS McIntyre, "X-ray photoelectron spectroscopy studies of reactions on chromium metal and chromium oxide surfaces," Journal of Electron Spectroscopy and Related Phenomena **184**, 29–37 (2011).

[4] Anil K Sinha and Kenichirou Suzuki, "Novel mesoporous chromium oxide for vocs elimination," Applied Catalysis B: Environmental **70**, 417–422 (2007).
